# Suprabasal Blistering as a Diagnostic Pitfall in Bullous Pemphigoid: Histopathologic-Immunofluorescence Discordance Complicated by Sepsis

**DOI:** 10.7759/cureus.115641

**Published:** 2026-09-02

**Authors:** Adriana V Saenz, Marisol Cazares, Sarahi G Romero, Mauro A Cardenas, Sofia de La O, Anna K Bustillos

**Affiliations:** 1 Internal Medicine, Hospital General “Presidente Lázaro Cárdenas del Río,” Instituto de Seguridad y Servicios Sociales de los Trabajadores del Estado (ISSSTE), Universidad Autónoma de Chihuahua, Chihuahua City, MEX; 2 Surgery, Hospital General “Presidente Lázaro Cárdenas del Río,” Instituto de Seguridad y Servicios Sociales de los Trabajadores del Estado (ISSSTE), Universidad Autónoma de Chihuahua, Chihuahua City, MEX

**Keywords:** autoimmune blistering disease, bullous pemphigoid, diagnostic pitfall, direct immunofluorescence, histopathologic discordance, immunosuppression complications, nosocomial sepsis, supra basal blister

## Abstract

Bullous pemphigoid is the most common autoimmune subepidermal blistering disease, typically diagnosed through a combination of clinical features, histopathology, and direct immunofluorescence (DIF). However, histopathological findings can be misleading when re-epithelialization shifts the blister plane from subepidermal to suprabasal, mimicking pemphigus vulgaris. We present the case of a 58-year-old woman with type 2 diabetes mellitus, diabetic neuropathy, and a sacral pressure ulcer admitted with a three-week history of generalized tense bullae in a symmetric distribution across the trunk and extremities, with subsequent oral mucosal involvement. The disease was classified as severe bullous pemphigoid. Skin biopsy revealed a suprabasal blister with eosinophilic infiltrate, a pattern classically associated with pemphigus vulgaris; however, DIF demonstrated linear immunoglobulin G and complement C3c deposition at the dermoepidermal junction, confirming bullous pemphigoid and categorically ruling out pemphigus.

Initial treatment with systemic corticosteroids and immunosuppressants was complicated by septic shock secondary to a catheter-related bloodstream infection, hospital-acquired pneumonia, and urinary tract infection, requiring vasopressor support, adjustment of the immunosuppressive regimen, and antimicrobial escalation. The patient also developed progressive multifactorial anemia, sepsis-associated coagulopathy, and upper gastrointestinal bleeding. Following multidisciplinary management, she achieved near-complete resolution of skin lesions and clinical recovery, and was discharged with plans for rituximab as definitive steroid-sparing therapy.

This case highlights two critical lessons: first, a suprabasal blister on histopathology does not exclude bullous pemphigoid, as re-epithelialization can alter the apparent cleavage plane, making DIF indispensable for an accurate diagnosis; second, severe bullous pemphigoid requiring intensive immunosuppression carries a substantial risk of sequential nosocomial infections warranting close microbiological surveillance, early source control, and timely modulation of immunosuppressive regimens.

## Introduction

Bullous pemphigoid is the most common autoimmune subepidermal blistering disease within the spectrum of pemphigoid disorders, predominantly affecting adults over 60 years of age [1]. It is mediated by IgG autoantibodies against the hemidesmosomal antigens BP180 and BP230, and is diagnosed through the combination of clinical presentation, histopathology, and direct immunofluorescence (DIF), the latter demonstrating linear IgG and C3 deposition at the basement membrane [2-4]. Mucosal involvement, most commonly oral, occurs in up to 20-30% of cases and is associated with greater disease morbidity [5].

Although a subepidermal cleavage plane is the classic histopathologic hallmark of bullous pemphigoid, this finding is not always straightforward. Re-epithelialization of evolving blisters can displace the apparent plane of cleavage from subepidermal to suprabasal, closely mimicking pemphigus vulgaris on light microscopy alone. Because histopathology alone can therefore be misleading, DIF is the decisive test that distinguishes between these two diseases and is indispensable for an accurate diagnosis - a diagnostic pitfall that is central to the case presented here.

Management of severe bullous pemphigoid requires systemic immunosuppression, most commonly high-dose corticosteroids, with adjuvant agents such as mycophenolate mofetil used in refractory or extensive disease [2,6]. This immunosuppression carries a significant risk of serious infection, with sepsis representing the leading cause of mortality in this population. Hospitalized patients with bullous pemphigoid also frequently present hematologic complications, including anemia related to chronic inflammation, cutaneous losses, and immunosuppressive-agent-induced myelosuppression, as well as comorbidities that further complicate management [7,8]. Balancing disease control against infectious and hematologic risk is therefore one of the greatest challenges in the inpatient management of severe bullous pemphigoid [2].

We present the case of a 58-year-old woman with severe bullous pemphigoid (Autoimmune Bullous Skin Disorder Intensity Score (ABSIS), 148 points) with oral involvement, in whom a suprabasal histopathological pattern initially raised suspicion for pemphigus vulgaris before DIF confirmed the diagnosis, and who subsequently developed septic shock from a central line-associated bloodstream infection during immunosuppressive treatment, complicated by multifactorial anemia. This case illustrates both the histopathologic diagnostic pitfall of bullous pemphigoid and the substantial infectious and hematologic risk of its immunosuppressive management in the critically ill patient.

## Case presentation

A 58-year-old Hispanic woman with a 16-year history of type 2 diabetes mellitus presented to the emergency department with a three-week history of progressive bullous skin lesions. Her medical history included diabetic neuropathy (diagnosed 2.5 years prior), grade 2 sacral pressure ulcer, and major depressive disorder managed for 10 years. She reported a penicillin allergy and denied tobacco or illicit drug use.

The skin lesions began approximately three weeks before presentation, starting as macules on the forearms that rapidly progressed to blisters and subsequently spread to the legs and perioral region. She also developed a single blister on the soft palate that ruptured, leaving an erosive lesion. The evolution followed a consistent pattern: initial blister formation, progression to flaccid bullae, rupture with subsequent painful ulceration, bleeding, and areas of necrosis particularly affecting the toes of both feet. She had initiated empirical treatment with erythromycin one week prior without clinical improvement.

Vital signs revealed fever (38°C), tachycardia (97 bpm), tachypnea (24 rpm), hypotension (97/65 mmHg), and hypoxemia (88% oxygen saturation on room air). Dermatologic examination showed generalized and disseminated dermatosis, predominantly affecting all four extremities with bilateral but asymmetric distribution. The lesions involved the lower extremities in their entirety and upper extremities (forearms, dorsum of hands and fingers), with additional involvement of bilateral upper eyelids, posterior cervical region, and perioral area. The scalp, face, and trunk were spared.

The morphology was polymorphic, characterized by flaccid bullae (3-5 mm to 5-6 cm in diameter) with serous to hemorrhagic content, multiple erosions with wound bed composed of granulation tissue, fibrin, and slough, ulcers with irregular and undermined borders, hemorrhagic and meliceric crusts, erythematous desquamative plaques, and dry necrosis involving the entirety of the left toes (Figures 1A, 1B, 1D) [1,2]. Nikolsky sign was positive. A single residual erosion was present on the soft palate without further mucosal progression. The clinical presentation at admission demonstrated extensive cutaneous involvement across multiple body regions, including facial lesions with perioral crusting (Figure 1E) [5], as well as an incisional biopsy site on the left forearm surrounded by active bullous lesions (Figure 1C).

**Figure 1:**
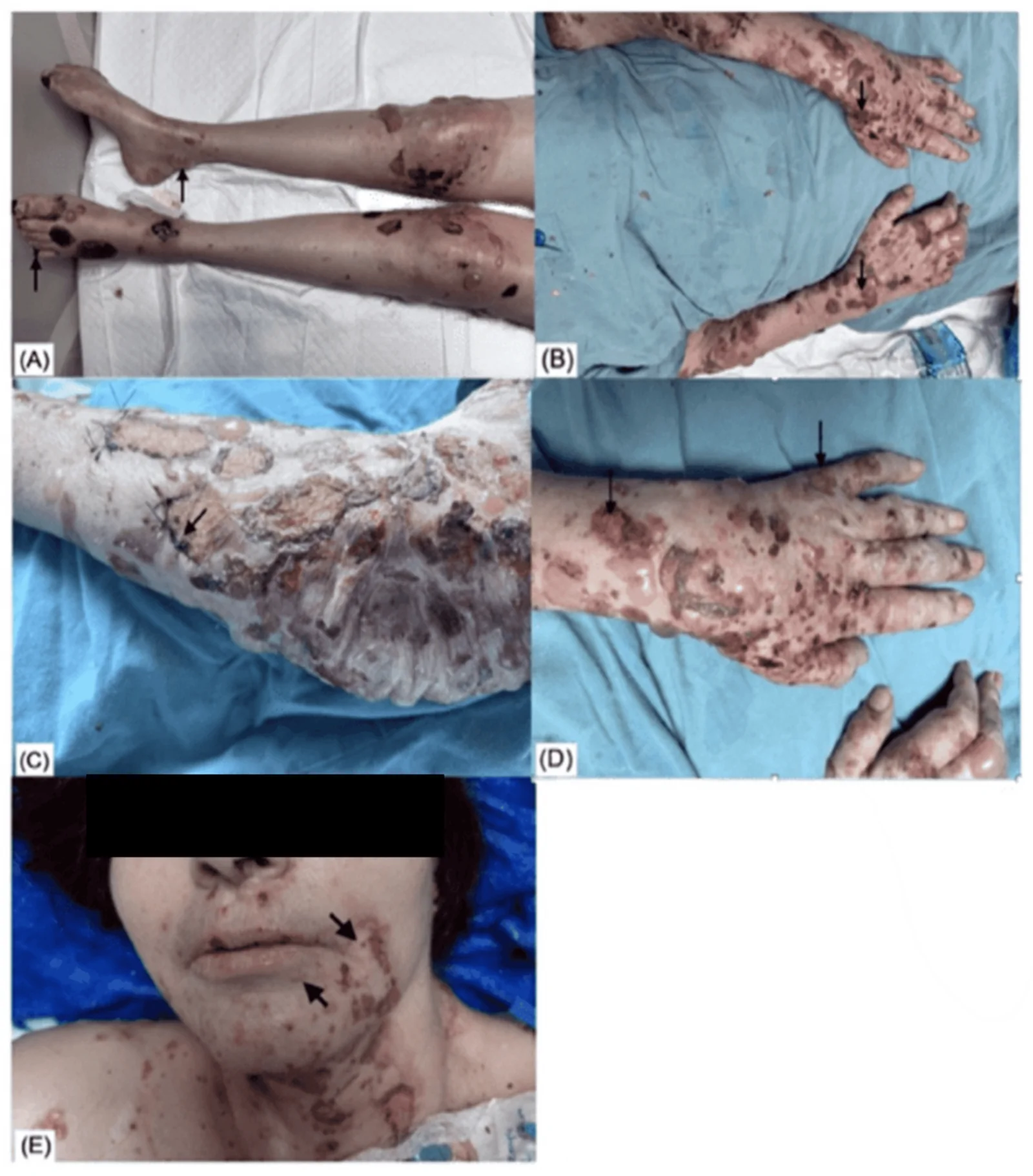
Clinical presentation at admission. (A) Lower extremities showing flaccid bullae, hemorrhagic bullae, erosions, and dry necrosis involving the left toes. Black arrows indicate representative flaccid bullae and dry digital necrosis. (B) Upper extremities showing multiple erosions, meliceric and hemorrhagic crusts on the dorsum of both hands and forearms. Black arrows indicate representative erosions and hemorrhagic crusts. (C) Left forearm with incisional biopsy site surrounded by active bullous lesions and crusts. The black arrow indicates the biopsy site. (D) Dorsum of the right hand showing confluent ulcers, meliceric crusts, and erythematous desquamative plaques. Black arrows indicate confluent ulcerations. (E) Facial involvement at admission showing perioral crusted lesions and scattered erythematous papules on the face and anterior chest. Black arrows indicate perineal crusted lesions. Written informed consent was obtained from the patient for publication of this case report and accompanying images.

Studies revealed microcytic hypochromic anemia (hemoglobin 10 g/dL (reference range: 12.0-15.5 g/dL), mean corpuscular volume (MCV) 79.1 fL (reference range: 80-100 fL)), leukocytosis (11,690/μL (reference range: 4,500-11,000 /μL)), elevated blood urea nitrogen (39.72 mg/dL (reference range: 7-20 mg/dL)), normal creatinine (1 mg/dL (reference range: 0.6-1.1 mg/dL)), and markedly elevated C-reactive protein (109 mg/L (reference range: <10 mg/dL)).

An incisional skin biopsy was performed on hospital day 3. Low-power microscopy showed a blister with suprabasal separation, with mild epidermal spongiosis. At higher magnification, the papillary dermis demonstrated a scattered eosinophilic infiltrate and capillary dilation (Figure 2). Given the suprabasal cleavage plane, the initial histopathologic differential diagnosis included pemphigus vulgaris and, less likely given the prominent eosinophilic infiltrate, IgA-mediated blistering disorders. However, DIF was required to confirm the diagnosis of bullous pemphigoid, given that a suprabasal cleavage plane can also be observed in this context.

**Figure 2:**
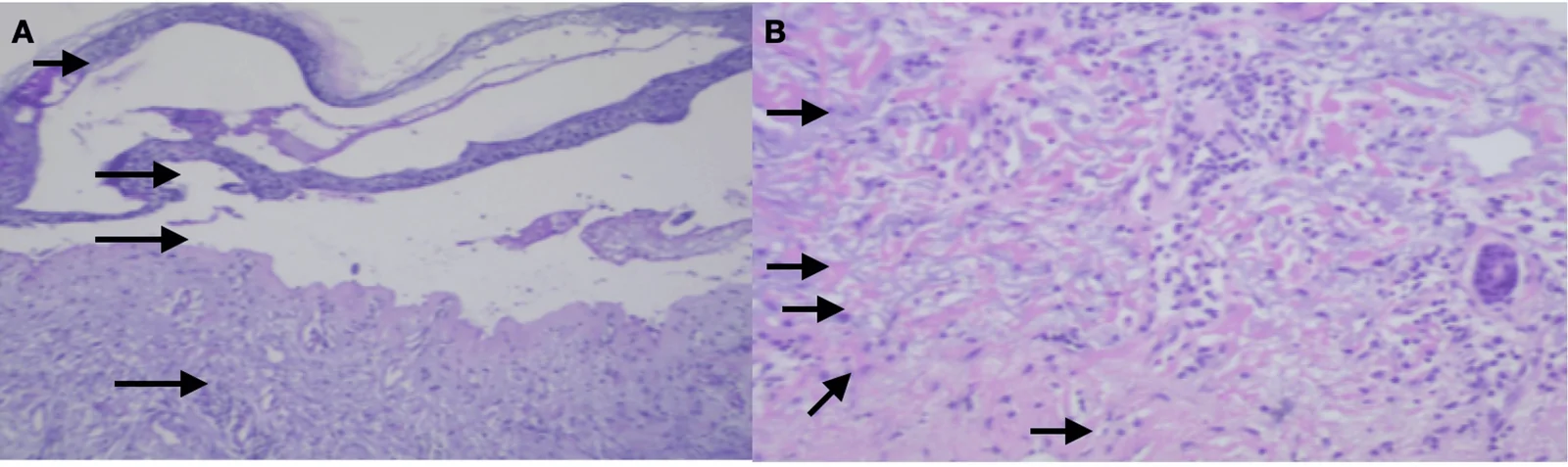
Histopathological findings. (A) Low-power view (H&E) showing a blister with suprabasal separation and mild epidermal spongiosis (black arrows). (B) High-power view demonstrating a scattered eosinophilic infiltrate and capillary dilation in the papillary dermis, findings compatible with pemphigus vulgaris; direct immunofluorescence was required to confirm the diagnosis of bullous pemphigoid (black arrows).

DIF microscopy was performed on perilesional skin to confirm the diagnosis. The study demonstrated strong (3+) linear IgG deposition at the base of the blister. Both IgA and IgG4 staining were negative. These findings are pathognomonic for bullous pemphigoid and confirmed the clinical and histopathologic diagnosis (Figure 3).

**Figure 3:**
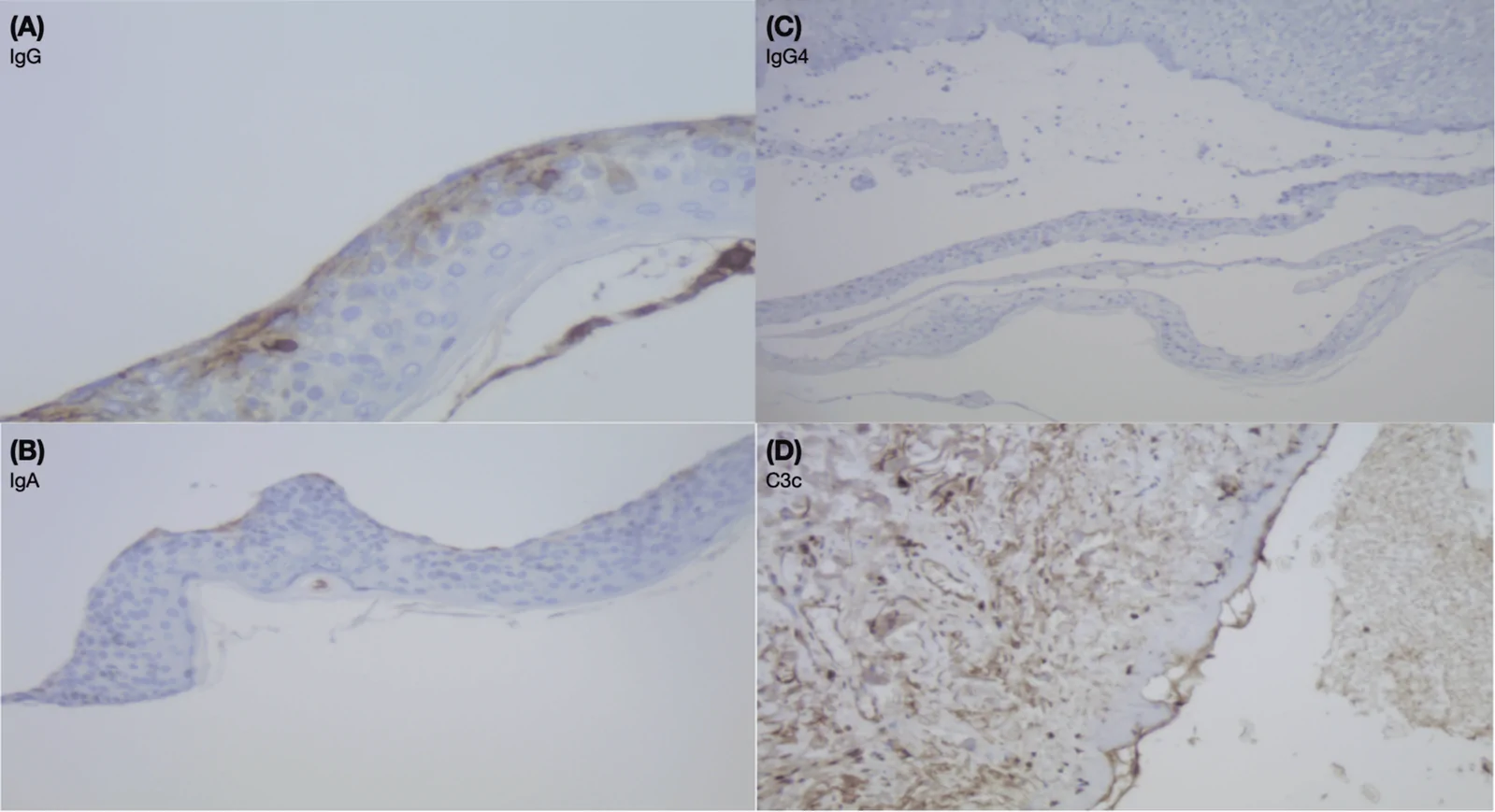
Direct immunofluorescence findings. (A) IgG: 3+ linear deposition along the basal layer of the epidermis. (B) IgA: negative. (C) IgG4: negative. (D) C3c: 3+ deposition at the base of the blister, confirming the diagnosis of bullous pemphigoid.

The diagnostic algorithm (Figure 4) illustrates the systematic approach based on immunofluorescence patterns to differentiate bullous pemphigoid from other autoimmune bullous diseases.

**Figure 4:**
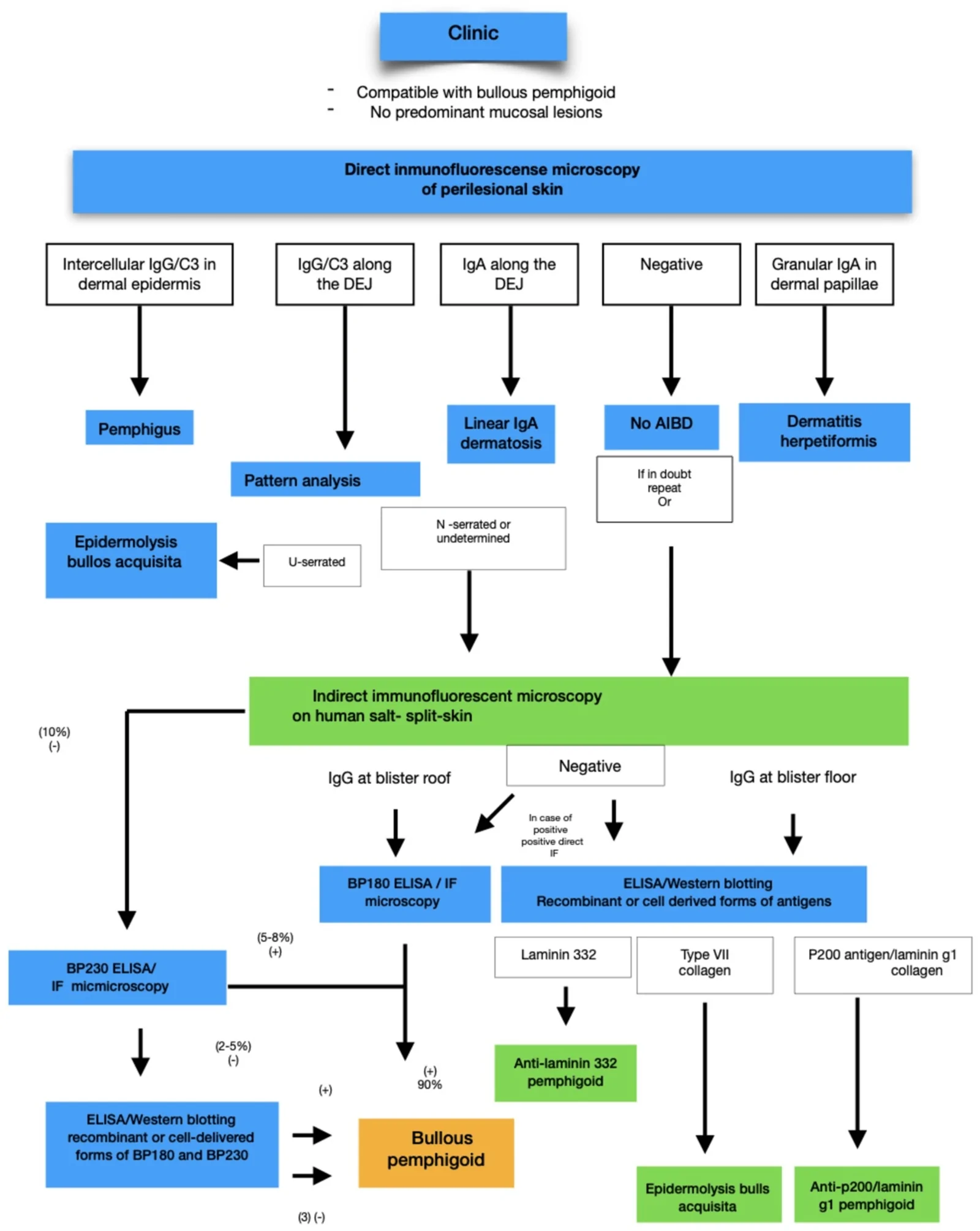
Diagnostic algorithm for bullous pemphigoid based on direct immunofluorescence microscopy patterns. The patient's biopsy demonstrated IgG/C3 deposition along the dermal-epidermal junction, consistent with the bullous pemphigoid pathway. DEJ: dermal-epidermal junction; Ig: immunoglobulin; C3: complement component 3; BP180/BP230: bullous pemphigoid antigens 180/230 kDa; AIBD: autoimmune bullous disease; ELISA: enzyme-linked immunosorbent assay; IF: immunofluorescence Original diagram created by the authors based on the diagnostic criteria and immunofluorescence patterns described in Rook's Dermatology Handbook [9].

Disease severity was assessed using both the Bullous Pemphigoid Disease Area Index (BPDAI) and the Autoimmune Bullous Skin Disorder Intensity Score (ABSIS), as the combined use of these validated indices provides complementary clinician-reported and patient-reported measures of disease burden [10,11]. The patient scored in the severe range on both scales (ABSIS 148 points), consistent with internationally validated severity thresholds [12]. Mortality risk factors included type 2 diabetes mellitus, Karnofsky functional status of 40%, and body surface area involvement greater than 30%.

Therapy was initiated with intravenous methylprednisolone (60 mg every 24 hours), broad-spectrum antibiotics (cefepime and clindamycin), and intensive wound care with copper sulfate, camphor, and zinc sulfate compresses followed by petrolatum application. Glycemic control was maintained with insulin glargine plus rapid-acting insulin on a sliding scale. Due to persistent disease extension with continued appearance of new bullous lesions, treatment was escalated on hospital day 7 to include intravenous immunoglobulin (IVIG) 2 g/kg (total dose calculated for 75 kg body weight, administered as 25 vials over two days), oral prednisone 0.75 mg/kg/day, and mycophenolate mofetil 500 mg every 12 hours.

On hospital day 20, the patient developed septic shock with multiple organ dysfunction. Blood cultures from central and peripheral lines grew *Staphylococcus haemolyticus* sensitive to vancomycin and linezolid. She developed hospital-acquired pneumonia (Figure 5) and pyelonephritis, requiring vasopressor support with norepinephrine (0.07 mcg/kg/min) and escalation of antibiotic therapy to vancomycin and meropenem. Her Sequential Organ Failure Assessment (SOFA) score was 6 points, and she met criteria for sepsis-induced coagulopathy with thrombocytopenia (platelets falling to 82,000/μL) and mildly prolonged coagulation times (prothrombin time (PT), 13.8 seconds; activated partial thromboplastin time (aPTT), 29.1 seconds).

**Figure 5:**
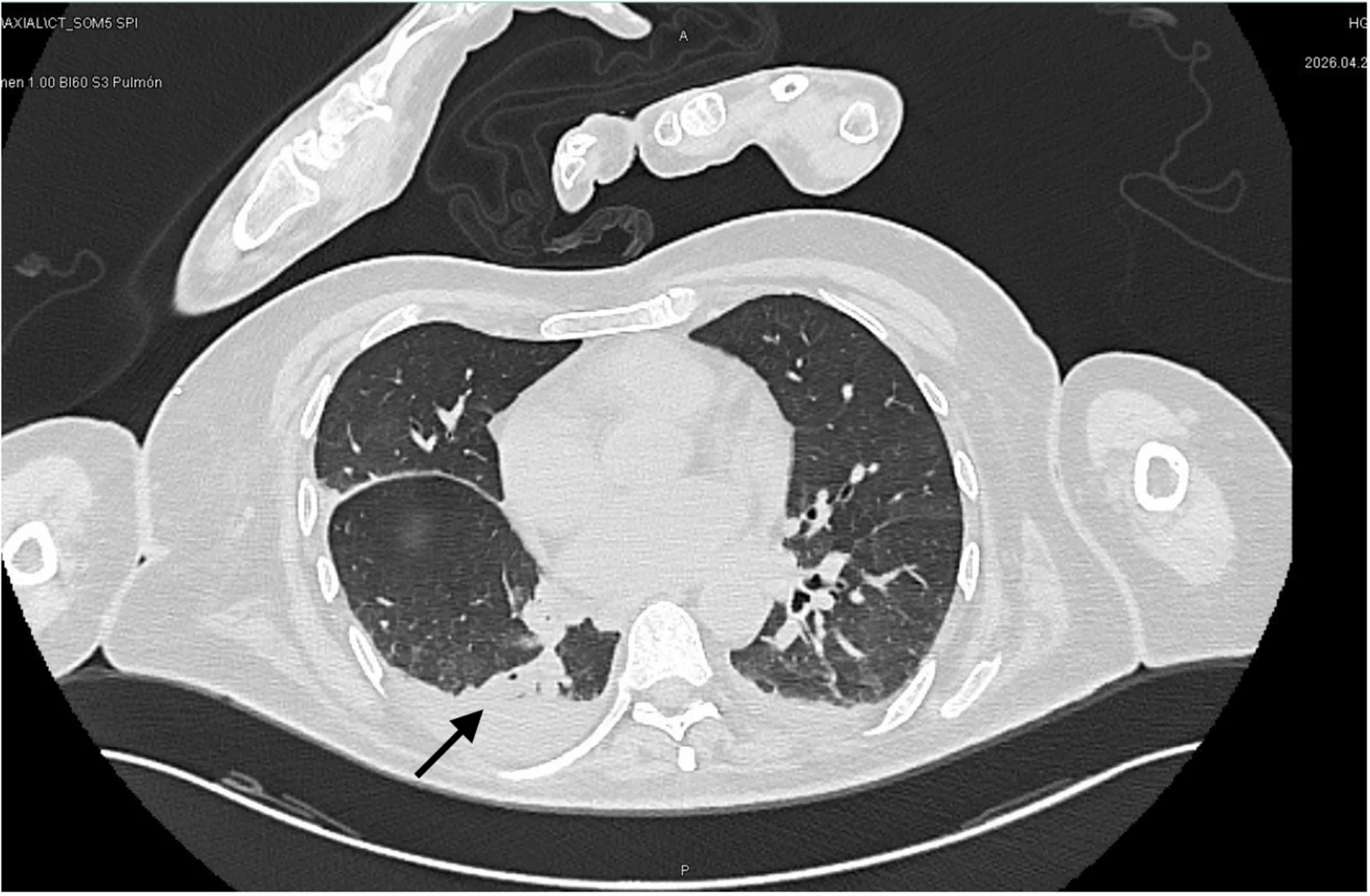
Chest computed tomography (CT) demonstrating hospital-acquired pneumonia (hospital day 20). Axial CT image in lung window showing left lower lobe consolidation with air bronchograms, ground-glass opacities in the left pulmonary field, and scattered nodular opacities in the right lung, consistent with hospital-acquired pneumonia (black arrow).

During the septic episode, oral prednisone was converted to intravenous hydrocortisone 200 mg/day (50 mg every six hours), approximately equivalent to prednisone 50 mg/day, providing stress-dose corticosteroid coverage while maintaining bullous pemphigoid disease control.

A subsequent central venous catheter tip culture grew *Burkholderia cepacia *complex, for which the catheter was removed and intravenous levofloxacin was administered for seven days.

The patient also developed progressive microcytic hypochromic anemia, with hemoglobin decreasing from 10 g/dL on admission to a nadir of 6.6 g/dL on hospital day 23. Hemolysis was excluded based on a negative direct Coombs test and normal lactate dehydrogenase (108 U/L). A low reticulocyte count (1%) confirmed an inadequate marrow response, consistent with drug-induced bone marrow suppression from mycophenolate. Additional possible contributors, not directly confirmed, included anemia of chronic disease/inflammation with iron sequestration, iron deficiency from cutaneous losses through extensive bullous lesions, and reduced oral intake due to oral lesions. Upper gastrointestinal endoscopy performed on hospital day 25 revealed chronic gastritis with positive fecal occult blood test. The patient required packed red blood cell transfusion, receiving one unit on hospital day 32, with post-transfusion hemoglobin increasing to 9.5 g/dL. Serum iron was markedly low at 13 mcg/dL (hospital day 1) and 21.9 mcg/dL (hospital day 34).

The patient demonstrated gradual clinical improvement with resolution of septic shock, successful weaning from vasopressor support by hospital day 25, and progressive healing of skin lesions without new blister formation by early May. Antibiotic therapy was de-escalated to vancomycin monotherapy on hospital day 29, completing a 14-day course. Intravenous hydrocortisone was transitioned back to oral prednisone 50 mg daily on hospital day 29 after resolution of septic shock.

By the time of hospital discharge on hospital day 35, the patient was hemodynamically stable with normalized vital signs. Physical examination showed significant improvement in skin lesions, with resolution of active blisters and presence of healing erythematous plaques with serosanguineous crusts, consistent with near-complete clinical response to treatment (Figure 6). The BPDAI score improved from the severe range on admission to 37 points (moderate range) at discharge, and the ABSIS score decreased substantially from 148 points to 13 points. The improvement in BPDAI exceeded the minimal clinically important difference (MCID) of 4 points for clinical improvement [13]. Laboratory values at discharge showed hemoglobin 10.6 g/dL (reference range: 12.0-15.5 g/dL), platelets 277,000/μL (reference range: 150,000-450,000/μL), white blood cells 7,270/μL (reference range: 4,500-11,000 /μL), and CRP 16 mg/L (reference range: <10 mg/L).

**Figure 6:**
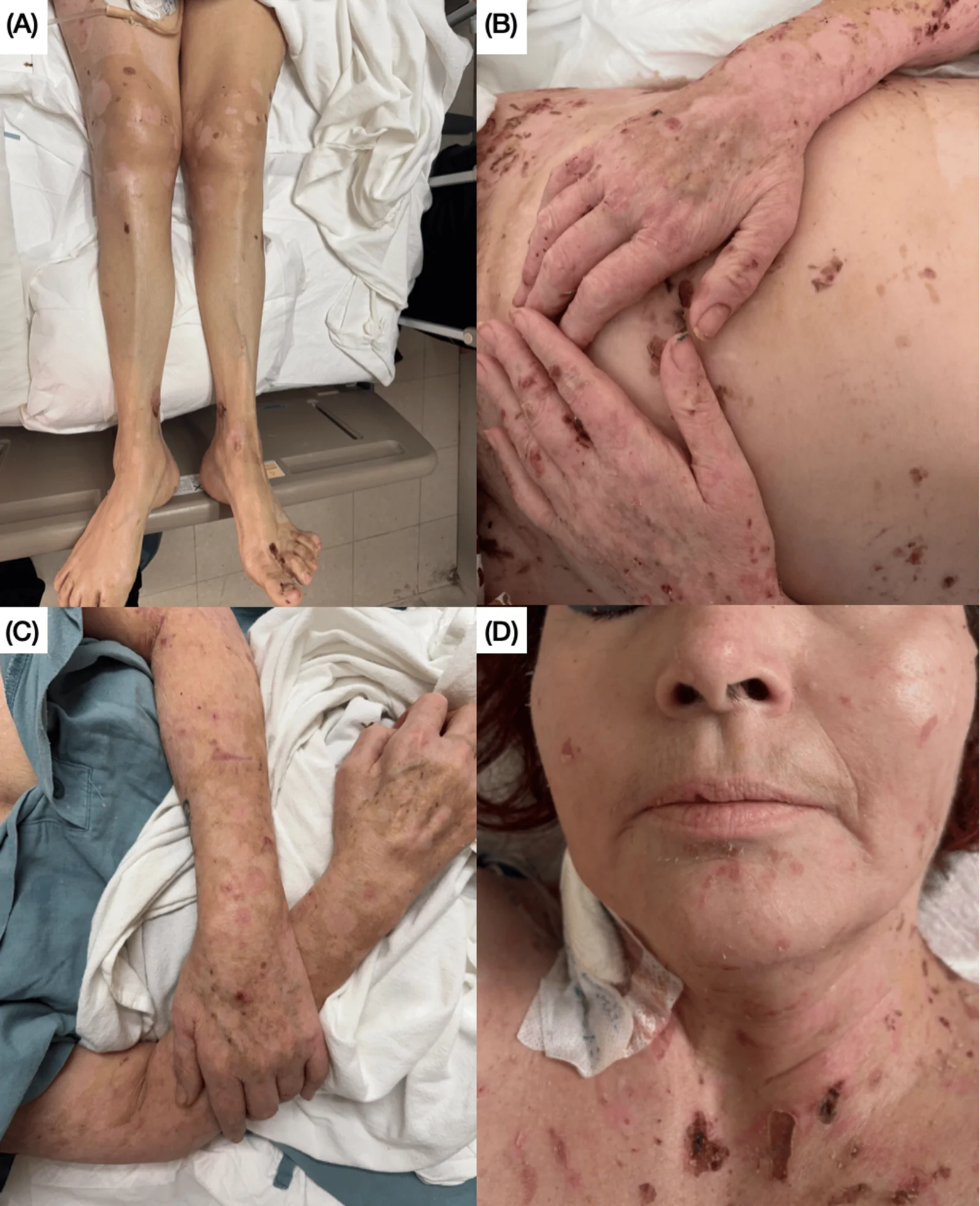
Clinical appearance at hospital discharge (day 35). (A) Lower extremities showing resolution of active bullae with healing erythematous plaques and residual crusted lesions. (B-C) Upper extremities and hands demonstrating significant re-epithelialization with residual serosanguineous crusts. (D) Facial region showing marked improvement with resolution of eyelid erosions and perioral lesions. Written informed consent was obtained from the patient for publication of this case report and accompanying images.

The patient was discharged with plans for outpatient administration of rituximab (1000 mg intravenous on days 1 and 15, following the rheumatoid arthritis (RA) protocol) as therapy for refractory bullous pemphigoid. Classification as refractory disease was based on the initial severe BPDAI score, extensive body surface area involvement (>30%), development of life-threatening septic complications during corticosteroid therapy, and the need for prolonged high-dose systemic corticosteroids with associated adverse events [3,14,15]. Rituximab administration was scheduled for one week after discharge, with consideration for restarting mycophenolate mofetil once the infectious process was completely resolved.

Discharge medications included prednisone 40 mg daily, insulin glargine 32 units daily with metformin for glycemic control, and topical wound care with Dalidome compresses followed by petrolatum and Dexeryl cream application two to three times daily. Additional medications for pre-existing comorbidities (neuropathic pain, hypertension, and psychiatric conditions) were continued at outpatient follow-up. A summary timeline of the key clinical events during hospitalization is presented in Table 1.

**Table 1:** Timeline of key clinical events during hospitalization. DIF: direct immunofluorescence; GI: gastrointestinal; BPDAI: Bullous Pemphigoid Disease Area Index; ABSIS: Autoimmune Bullous Skin Disorder Intensity Score; IV: intravenous; IVIG: IV immunoglobulin

Hospital day	Event/finding	Action taken
Day 1 (admission)	Generalized tense bullae, erosions, and necrosis; fever, tachycardia, hypotension, hypoxemia	Admission; initial workup
Day 3	Incisional skin biopsy performed	Histopathology sent
Days 3-10	Suprabasal blister with eosinophilic infiltrate reported; DIF pending	IV methylprednisolone (60 mg/24h) initiated
Day 7	Persistent disease extension with new bullous lesions	Escalation to IVIG (2 g/kg over two days), prednisone 0.75 mg/kg/day, and mycophenolate mofetil 500 mg/12h
Day 13	DIF confirmed bullous pemphigoid (linear IgG 3+, C3c 3+)	Diagnosis confirmed
Day 20	Septic shock, multiple organ dysfunction; catheter-related bacteremia (*Staphylococcus haemolyticus*), hospital-acquired pneumonia, pyelonephritis	Vasopressor support (norepinephrine); antibiotics escalated to vancomycin + meropenem; oral prednisone converted to IV hydrocortisone
Day 23	Hemoglobin nadir (6.6 g/dL)	Anemia workup initiated
Day 25	Vasopressor weaning completed; upper GI endoscopy revealed chronic gastritis	Endoscopy performed
Day 29	Sepsis resolved	Antibiotics de-escalated to vancomycin monotherapy (14-day course); IV hydrocortisone transitioned back to oral prednisone 50 mg/day
Day 32	Persistent anemia	Packed red blood cell transfusion (one unit)
Day 35	Near-complete clinical response (BPDAI 37, ABSIS 13)	Discharge with plans for outpatient rituximab

## Discussion

Bullous pemphigoid in younger patients: an atypical presentation

Bullous pemphigoid predominantly affects elderly patients, with a median age of presentation in the seventh to eighth decade of life and onset usually in the late 70s [2,16]. However, bullous pemphigoid can also affect younger individuals, though this is uncommon, with an incidence of less than 0.5 cases per one million people per year in those younger than 50 years [16]. This case illustrates a 58-year-old patient with severe bullous pemphigoid, representing an atypical age of presentation that posed unique management challenges, particularly when complicated by life-threatening infections. The patient's ABSIS score of 148 points classified her disease as severe, requiring aggressive immunosuppressive therapy that paradoxically increased her susceptibility to infectious complications.

Recent evidence suggests that younger patients with bullous pemphigoid may present with more severe disease manifestations. A study by Chen et al. demonstrated that patients under 60 years had higher anti-BP180 IgE titers, increased C3 deposition, higher hospitalization rates, and significantly elevated serum concentrations of pro-inflammatory cytokines (interleukin-13 (IL-13), tumor necrosis factor-alpha (TNF-α), and interferon-gamma (IFN-γ)) compared to elderly patients [17]. Nearly 52% of patients under 60 were misdiagnosed on their first visit, often presenting with oral mucosal involvement [17].

This patient presented with multiple risk factors including diabetes mellitus and severe disease requiring high-dose systemic corticosteroids, placing her at substantially elevated risk for both infectious complications and mortality. Serious infections are the leading cause of death in patients with bullous pemphigoid [18,19]. Studies demonstrate that bronchopneumonia and infectious diseases are among the most common causes of death in this population [19]. Systemic corticosteroid use has been identified as the strongest risk factor for serious infections, resulting in a two-fold increase in infection risk [19].

This case exemplifies this high-risk scenario: the patient developed polymicrobial bacteremia and septic shock while receiving prednisone 50 mg/day, requiring ICU admission and broad-spectrum antimicrobial therapy. Multiple studies have identified specific risk factors that predict mortality and infectious complications in bullous pemphigoid patients (Table 2). This patient presented with several high-risk features including diabetes mellitus, severe disease, high-dose systemic corticosteroid use, and hypoalbuminemia.

**Table 2:** Risk factors for mortality and infectious complications in bullous pemphigoid. BP180: Bullous Pemphigoid antigen 180

Risk factor	Impact	Hazard ratio (HR)/odds ratio (OR)	References
Diabetes mellitus	Increased mortality	HR 2.39 (1.55-3.69)	[2]
Diabetes mellitus	Increased infection risk	OR 2.67 (1.44-4.95)	[18]
High-dose systemic corticosteroid use	Increased infection risk	OR 2.54 (1.46-4.43)	[18]
Hypoalbuminemia	Increased infection risk	OR 2.56 (1.28-5.09)	[18]
Hospitalization	Increased infection risk	OR 4.03 (2.29-7.08)	[18]
Respiratory disease comorbidity	Increased infection risk	OR 4.06 (1.86-8.86)	[18]
Positive BP180 antibody	Increased mortality	HR 1.85 (1.25-2.75)	[2]
Heart disease	Increased mortality	HR 1.96 (1.41-2.73)	[2]
Stroke	Increased mortality	HR 2.09 (1.23-3.55)	[2]
Dementia	Increased mortality	HR 2.26 (1.43-3.59)	[2]
Advanced age (>78-80 years)	Increased mortality	Variable by study	[6,17]

Corticosteroid management during sepsis

The management of immunosuppressive therapy during active infection presents a clinical dilemma. While abrupt discontinuation of corticosteroids in patients on chronic therapy can precipitate adrenal crisis, continuation of high-dose immunosuppression may impair the host immune response to infection. In this case, converting oral prednisone to intravenous hydrocortisone 200 mg/day (50 mg every six hours), approximately equivalent to prednisone 50 mg/day, served a dual purpose: it provided stress-dose corticosteroid coverage for septic shock in accordance with the 2021 Surviving Sepsis Campaign guidelines, while simultaneously maintaining a therapeutic dose equivalent for ongoing bullous pemphigoid control.

This approach represents a pragmatic balance between preventing adrenal insufficiency and avoiding excessive immunosuppression during critical illness. In this case, sepsis resolved with this regimen combined with appropriate antimicrobial therapy and supportive care; however, the individual contribution of the corticosteroid strategy to this outcome, relative to antimicrobial therapy and supportive care, cannot be established from a single case.

Steroid-sparing strategies: lessons for future management

The high risk of infectious complications associated with systemic corticosteroids underscores the importance of steroid-sparing strategies in bullous pemphigoid management [2]. For patients with widespread disease, lower doses of oral prednisolone (0.5 mg/kg/day) are adequate for disease control and reduce adverse effects compared to higher doses [2]. Emerging evidence supports the use of doxycycline 200 mg/day as a safer alternative to oral prednisolone, with acceptable blister control and reduced treatment-associated mortality [6].

Additionally, newer biologic therapies, including rituximab, have demonstrated efficacy as steroid-sparing agents in refractory bullous pemphigoid [14,15,19], and JAK inhibitors (abrocitinib) combined with corticosteroids have shown promise in achieving faster disease control and more rapid corticosteroid tapering compared to conventional azathioprine-based regimens.

The rituximab dosing protocol adapted from the RA indication (1,000 mg on days 1 and 15) is the most commonly used strategy for bullous pemphigoid and has demonstrated superior efficacy in achieving complete remission compared to alternative regimens, including the lymphoma protocol (375 mg/m^2^ weekly for four weeks) and ultra-low-dose regimens (100 mg weekly for four weeks) [14-16]. Systematic reviews report complete remission in 70.5% of patients with refractory bullous pemphigoid within a median of 5.7 months, with partial remission in an additional 23.8% [14]. When used as initial adjuvant therapy combined with corticosteroids, rituximab significantly reduces time to complete remission (median 215 days versus 529 days with corticosteroids alone) and provides substantial corticosteroid-sparing effects [16]. The recurrence rate after rituximab therapy is approximately 20.5%, with infection as the most common adverse event (6.6%) [14]. In elderly patients with refractory disease, rituximab has demonstrated favorable safety and long-term outcomes [14-16]. As rituximab had not yet been administered by the time of this report, its actual efficacy and safety in this specific patient could not be assessed.

In retrospect, earlier initiation of steroid-sparing agents or consideration of alternative therapies might have reduced this patient's infection risk, though the severity of her presentation necessitated aggressive initial immunosuppression. This is particularly relevant in younger patients with bullous pemphigoid, who may have more severe disease manifestations and higher inflammatory cytokine profiles [17].

Clinical implications

This case highlights the importance of recognizing that bullous pemphigoid in patients younger than the typical bullous pemphigoid population may present with more severe disease manifestations, higher inflammatory cytokine profiles, and comorbidities such as diabetes mellitus, warranting heightened clinical vigilance in this subgroup [17-20].

The successful management of this patient's septic episode, followed by her eventual recovery and discharge, demonstrates that even critically ill patients with bullous pemphigoid complicated by severe infections can achieve favorable outcomes with aggressive supportive care, appropriate antimicrobial therapy, and judicious immunosuppressive management.

## Conclusions

This case illustrates critical challenges in the management of severe bullous pemphigoid in a patient with a relatively young age of onset (58 years) and significant comorbidities. First, it illustrates that the presence of a suprabasal blister on histopathology does not rule out bullous pemphigoid; re-epithelialization is a recognized mechanism that can displace the apparent cleavage plane and mimic pemphigus vulgaris, although this was not directly demonstrated on pathology in this case. DIF remains essential to confirm the diagnosis regardless of the histopathologic cleavage plane observed.

Second, this patient's development of polymicrobial bacteremia and septic shock while on high-dose corticosteroids illustrates the well-documented risk of serious infection as the leading cause of mortality in this population. Her clinical course highlights the difficulty of individualizing immunosuppressive therapy during active infection, balancing the risks of adrenal insufficiency, disease reactivation, and impaired immune response. This case underscores the need for heightened vigilance for sepsis in patients with bullous pemphigoid who present with diabetes mellitus, hypoalbuminemia, or severe disease, particularly during high-dose immunosuppressive therapy, and suggests that earlier consideration of steroid-sparing strategies may merit further evaluation in similarly high-risk patients.
